# Therapeutical and Pharmaceutical Notes

**Published:** 1899-08

**Authors:** W. J. Martin

**Affiliations:** Kanakee, Ill.


### THERAPEUTICAL AND PHARMACEUTICAL NOTES.

Under the Direction of W. J. Martin, M.D.C.,
KANAKEE, ILL.

A Treatise on Surgical Therapeutics of Domestic Animals. We have received Part I. of Vol. I. of the work entitled, *A Treatise on Surgical Therapeutics of Domestic Animals*, by Profs. P. J. Cadiot and J. Almy, of the Veterinary School of Alfort. The work is being translated into English by Prof. A. Liautard. It should be a matter of congratulation to the English-speaking veterinary world that such a veteran veterinarian as Dr. Liautard has seen fit to place before them in his most masterly and lucid style such an exhaustive work as this one is on one of the most important branches of our profession.

Heretofore in veterinary literature standard works devoted exclusively to veterinary surgery and surgical therapeutics have been all too few in number, and although these works were fairly complete in the grouping of the various subjects upon which they treated, their very scarcity in number often compelled the veterinary practitioner and advanced student to seek fuller scientific knowledge of surgical technique in the numerous and more exhaustive treatises on human surgery. The advent, then, of this important work is most timely, and will mark a new era in the advance of English veterinary literature.

Chapter I. of the *Treatise* is devoted to a description of the various means of restraint employed in securing the different species of domestic animals for surgical operations. Illustrations of many of these appliances are shown in excellent woodcuts.

Chapter II. contains a summary of the methods employed in procuring general anæsthesia among domestic animals. This chapter is most complete in every detail, treating of the *modus operandi* of anæsthesia. It begins by giving a short historical account of the discovery of anæsthesia by Warren, of Boston, Mass., in 1846, when before a class of medical students he demonstrated that after the inhalation of ether, surgical operations could be performed in a perfectly painless manner. In the following year general anæsthesia was introduced into the veterinary profession by Renault, Bouley, and others. The section treating on the various operations in which general anæsthesia is indicated, and of the numerous physiological or pathological changes that may be present in an organism which would contraindicate the administration of an anæsthetic are very complete in detail, and bear every evidence of having been prepared with great care, and by operators entirely familiar with the actions and uses of the many therapeutical agents used to produce anæsthesia among domestic animals. A long list of these drugs is included in this chapter, together with the names of each species of animals for which the drugs can be most appropriately used for anæsthetic purposes.

Chapter III. opens with a dissertation on surgical antisepsis and asepsis, giving a short *résumé* of the surgical dressing of wounds so as to exclude atmospheric germs, the infectiousness of which surgeons it appears were aware of even in remote times. There is, perhaps, no chapter in any work extant on veterinary surgery in the English language that will prove of greater interest to the progressive veterinarian than the one treating on antisepsis and asepsis in this treatise. This subject is especially well handled, and, in addition, contains an interesting table showing the different temperatures at which the various micrococci and bacilli are destroyed or their toxic effects rendered harmless by heat. A very complete list of antiseptic drugs is given, together with working formulæ for preparing the various antiseptic solutions for surgical work.

Chapter IV. treats of surgical hæmostasis, and gives the latest methods for arresting or controlling hemorrhage during surgical manipulations. This chapter will be found to be of especial value to all operating surgeons.

Chapter V. contains a detailed description of the methods employed in cauterization, or firing, together with many illustrations showing the many different instruments used for this purpose. A section is also devoted to the best means to be employed in treating parts to which the cautery has been applied.

Taken as a whole, the work may be considered a valuable acquisition to modern veterinary literature. We bespeak for it a cordial welcome by American veterinarians, to whom, no doubt, it will become an indispensable work of reference.

The work is to be issued in parts, and the entire set will form two large volumes. It is published by W. R. Jenkins, of New York.

RHEUMATIC LINIMENT. ℞.—Spirit of camphor, tincture of opium, aqua ammonia, of each ʒij.; olive oil ʒiv. M.—*Merck's Archives.*

PRESCRIBING IODIDE POTASSIUM. Dr. Kœning holds that the best method of administering potassium iodide is in the form of an aqueous solution, in which one drop represents approximately one grain of salt. To prescribe the two, ounce for ounce, results in a solution measuring eleven fluidrachms, and one drop of this necessarily contains considerably less than one grain of the iodide. To overcome this Professor Hynson makes the following suggestion: Dissolve 480 grains of salt in five-and-a-half drachms of hot water, and then make up the solution to eight drachms with water. This always results in a solution representing one grain to each minim, and approximately, in each drop.

Administered in this form, in gradually increased doses, the severe symptoms of iodism may always be prevented, nor is the concentration of the solution a contraindication against the addition of corrosive sublimate, should it be desired to administer the mercury in the form of a biniodide.—*Medical Standard.*

AN EFFECTIVE REMEDY. According to recent press dispatches, a farmer living near Zurich, Ont., in order to rid his cattle of lice in a cheap and effective manner, saturated the skin of the animals with kerosene oil, and to one of these, a young heifer, he applied a lighted match with the most surprising results. The man himself was severely burned, his barn and its contents entirely consumed, as were also several head of valuable cattle.

Is it any wonder that the fool-killer is overworked in this world?

EXPLOSIVE FORMALDEHYDE MIXTURE. *The Pharm. Gen. Anzeiger* reports that when formaldehyde, nitric acid, and volatile oils are mixed, heat is quickly generated and an explosion ensues. It is well to remember this reaction.

ETIOLOGY OF DYSENTERY. In the *Supplemento al Policlinico*, of February 11, 1899, Professor Celli and Dr. Valenti, of Rome, published an important note on the "Etiology of

Dysentery." The authors stated that, in 1896, Professor Celli published *in extenso*, in the *Annali d'Igiene Experimentale*, his remarks on the "Etiology of Dysentery in its Relation with the Bacillus Coli and with its Toxins," in which he demonstrated that the diagnosis of the bacteria can be made by the study of the action of their toxins, and by this means he differentiated from the many varieties of the bacillus coli that form which he called the bacterium colidysentericum, because he considered it the specific cause of dysentery in man. These researches were afterward confirmed by Del Pino and by Alessandri. Since then the authors have continued their experimental observations on the toxic products of the same variety of bacteria, and in the respective antitoximmunity and antitoxitherapy. Using the toxin obtained from alcoholic precipitation of both cultures filtered through blotting-paper, and reduced to powder toxiprotein—that is, a mixture of toxin and protein—after a long series of useless attempts to immunize dogs, they, on May 4, 1897, succeeded in immunizing an ass, at first subcutaneously and then endorenously. This animal after a long time no longer reacted to the toxic inoculation, so that they gradually arrived at from seven centigrammes of dry toxin injected subcutaneously to one gramme injected into the veins. The serum obtained they call, for brevity and clearness of explanation, Serum A.

Meanwhile Valenti studied the action of the protein of the bacillus colidysentericum, and comparatively of other bacillus coli, extracting them according to Koch's method for the new tuberculin. He demonstrated that they had no action in herbivora, but instead a characteristic action in dogs and cats, chiefly in the large intestine, and analogous to that of the above-mentioned toxiprotein. With these two proteins, which they call C. O. and C. R., they tried to immunize two young asses. Of these two animals that inoculated with C. O. died of very acute intoxication after the third injection and a dose of C. O., c.c. 18=38 millegrammes of dry substance. They, therefore, had to immunize with the same C. O. another young ass. They call Serum B that obtained from the animal inoculated with C. R., and Serum C that extracted from the animal injected with C. O. The three animals, inoculated respectively with dry toxiprotein, with C. R., and with C. O., are still undergoing the injection of the same toxic substances in the Milan Serumtherapic Institute.

The authors then give particulars of some of their experiments on kittens to demonstrate the degree of immunity reached and the value of the respective serums. The results obtained are that Serum A is the most efficacious both as a preventive and curative; that Serum B and C act as preventatives, but as curatives they have uncertain (Serum B) or no action (Serum C), succeeding only in retarding death for a few days. Death took place by marasmus without any apparent organic lesions or intestinal localization, which demonstrated that in the body of this bacillus coli dysentericum, as in many other pathogenic and saprogenic bacteria, a marantic toxin is found, which, inoculated into animals, does not produce any counter-poison.

The authors note that the results of analogous experiments were not always equal to the preceding, and those in which they lessened the quantity of the serum were negative; so that for dysentery they were very far from those high degrees of active immunity, and the consequent passive immunities, that are obtained against distinctly toxic infections, such as diphtheria and tetanus. With the best of their serums—that is, Serum A—they experimented on the serum diagnosis of several bacilli coli in the bacteriological laboratory of the Rome Institute of Hygiene. The results obtained with the hanging drop confirmed the specificity of the Serum A in respect to the bacillus coli dysentericum, and also confirmed the difference from the other bacteria of the same species.

During the last summer, an epidemic of dysentery being prevalent in the Undine, the authors tried the serum-therapy. Having only a few cases at their disposal they wished to test the three serums, first inoculating subcutaneously Serum A, and then the others. The results were as follow: Of six cases of acute dysentery, recently developed, with the serum treatment there was a rapid disappearance in all in two to five days of the sanguinolent feces, and a ready cure. In a seventh case of twenty days' duration, in an old woman of eighty, also suffering from mitral insufficiency and parenchymatous nephritis, the serum-therapy had no action. At the same time, of four other patients admitted to the hospital suffering from acute recent dysentery, and treated in the ordinary way, three died. The authors state that these result have encouraged them to continue the immunization of their animals to prepare the serum for testing on a large scale.

Recently Shiga, of Kitasato's Institute for Infectious Diseases, has published a work in which he confirms the fact that the cause of dysentery is a variety of the bacillus coli, which he has differentiated by means of serum-diagnosis, that is, with the serum of the blood of persons suffering from dysentery. To solve the doubt that Shiga has raised as to the identity of his bacillus dysentericus with the bacterium dysentericum (Celli), the authors retested and compared its cultural characters, and they found that after many successive passages through animals and in cultures for preserving or exalting the virulence it maintains a typosimile, as in the bacillus dysentericus. The authors then describe the appearance in gelatin stab-cultures, and the results of the serum-diagnosis with the blood of dysenterics, and they have come to the conclusion that, according to every probability, the bacterium colidysentericum (Celli) is identical with the bacillus dysentericus (Shiga).—*Therapeutic Gazette.*

---

New York State continues to give public exhibitions of the value of tuberculin as a diagnostic agent in bovine tuberculosis to the edification of junketing commissions and their friends of the State Legislature. Surely this period of inquiry has passed, and the results fixed and definite, and it is quite time that the Empire State was doing more to relieve their people of the continued danger from this source, and their live-stock owners and breeders in greater measure from the continued drain of their resources from this insidious disease.

A correspondent, in speaking of his experience in the work of the Bureau of Animal Industry, says: "I have found it somewhat more interesting than at first, and surely one can learn many things in gross pathological anatomy, and from diseases and diseased conditions found both ante- and post-mortem. I can more and more see the necessity of a thorough meat-and-milk inspection system. Not alone for our inter-State and export trade, but at home as well; but, of course, to accomplish this the force must be increased, laws passed, and the people made to see the importance of such a step; to say the least it will be no easy task, but it is inevitable, and we might just as well prepare ourselves for it, put our shoulders to the wheel, and push it along."

